# Deep learning for detecting depression in individuals with and without alexithymia

**DOI:** 10.1038/s43856-026-01393-0

**Published:** 2026-01-16

**Authors:** Calvin Lam, Longdi Xian, Rong Huang, Jie Chen, Kit Ying Chan, Joey W. Y. Chan, Steven W. H. Chau, Ngan Yin Chan, Shirley Xin Li, Yun-Kwok Wing, Tim M. H. Li

**Affiliations:** 1 Li Chiu Kong Family Sleep Assessment Unit, Department of Psychiatry, The Chinese University of Hong Kong, Hong Kong, China; 2 Department of Psychiatry, Fujian Medical University Affiliated Fuzhou Neuropsychiatric Hospital, Fuzhou, China; 3 Department of Psychology, The University of Hong Kong, Hong Kong, China; 4 The State Key Laboratory of Brain and Cognitive Sciences, The University of Hong Kong, Hong Kong, China; 5 Li Ka Shing Institute of Health Sciences, Faculty of Medicine, The Chinese University of Hong Kong, Shatin, Hong Kong China

**Keywords:** Depression, Medical research

## Abstract

**Background:**

To accurately detect individuals’ mental health issues using artificial intelligence and self-report scales, it is crucial to recognize how personal characteristics can affect the detection. This study focuses on the role of alexithymia—a condition where individuals struggle to recognize and articulate emotions and symptoms—in the detection of depression. We aimed to determine whether deep learning models could enhance the accuracy of depression detection in people with alexithymia compared to self-report scales.

**Methods:**

We analyzed data from 194 patients with major depressive disorder and 105 community controls, employing eight large language models (LLMs) trained on transcript text from clinician-administered structured interviews using the Hamilton Depression Rating Scale (HAMD).

**Results:**

Here we show that generalized logistic regression analysis indicates a positive relationship between alexithymia and depression. Using the HAMD as the gold standard, individuals with alexithymia show poorer performance on the self-reported Hospital Anxiety and Depression Scale–Depression Subscale (HADS-D) in identifying depression (b = −0.37, p = .002). Four of the eight LLMs (AUCs=0.87-0.89) significantly outperform the HADS-D (AUC = 0.79) in depression detection (p <0.05). Subgroup analysis demonstrates that while LLMs achieve AUCs ranging from 0.79 to 0.96, the HADS-D only reaches an AUC of 0.35 for individuals with alexithymia.

**Conclusions:**

Our findings reveal that LLMs can potentially outperform self-report scales in detecting depression, particularly in those with alexithymia. These results highlight the importance of considering patient characteristics, such as alexithymia, when detecting depression. Deep learning analyses can enhance the accuracy of clinical assessments for depression and potentially for other mental health disorders.

## Plain language summary

People with alexithymia, who have difficulty identifying and expressing their emotions, may inaccurately report depression on self‑administered questionnaires. Artificial intelligence (AI) might help. We compared self-reports and eight AI models to detect clinician-rated depression from interview transcripts in people with and without alexithymia and major depression. Results indicate that alexithymia is associated with depression. The self-report scores are much less accurate for people with alexithymia, whereas all AI models detect depression more reliably in those individuals. Additionally, four AI models outperform the self-report across people with and without alexithymia. AI has the potential to improve depression detection among people who struggle to describe their emotions, warranting further research into other mental health disorders.

## Abstract (web summary)

Lam et al. evaluate a self-report scale and eight BERT-family large language models trained on clinical interview transcripts to detect clinician-rated depression in people with and without alexithymia. All models detect depression more accurately than the self-report scales in detecting depression, particularly in those with alexithymia.

## Introduction

Depression, a widespread mental health condition, is commonly evaluated through clinical interviews and self-report scales. While self-report scales, such as the Patient Health Questionnaire-9 (PHQ-9)^1^ and the Hospital Anxiety and Depression Scale–Depression Subscale (HADS-D)^2^, are widely used for their efficiency and accessibility, they may not be accurate for everyone, particularly for individuals with alexithymia^3,4^. Alexithymia, a psychological condition affecting 9–10% of the population^5^, involves difficulty in identifying and expressing emotions, which can hinder accurate self-reporting of depressive symptoms^3–6^. In contrast, clinical interviews are more reliable as they incorporate professional assessments and allow clinicians to ask follow-up questions^7^. This interactive approach helps clarify symptoms and provides a more accurate detection of depression, especially when patients struggle to effectively self-report.

In clinical interviews, patients express their symptoms verbally, allowing clinicians to ask questions and collect sufficient information to make judgments regarding the presence of depression^7^. This process provides rich textual data that can be leveraged for more comprehensive clinical assessments and text analysis. Linguistic features can significantly aid in recognizing emotions and mental health symptoms^8,9^. In recent years, researchers have explored digital assessment approaches to enhance depression detection, including artificial intelligence (AI) algorithms and large language models (LLMs)^10,11^. Researchers have employed LLMs to analyze text data for depression detection^12^. Models such as BERT, XLNet, RoBERTa, ALBERT, ELECTRA, and DistilBERT, along with domain-specific models like MentalBERT, have shown promising results in detecting mental health issues across various data sources, including electronic health records, social media posts, and clinical interviews^13^.

A significant issue arises from the frequent use of self-report scales as the gold standard for developing and validating AI-based depression detection models^14,15^. This reliance can be problematic because self-report scales may not be accurate for everyone, particularly for individuals with alexithymia—a condition marked by difficulty in identifying and expressing emotions, which can impair self-reporting of depressive symptoms^3–6^. However, limited research has explored how such patient characteristics may affect the accuracy of AI-based detection and self-report scales, revealing a research gap in understanding how alexithymia might diminish the effectiveness of these tools in accurately detecting depression. To investigate this question, the current study specifically focuses on detecting depression among individuals with and without alexithymia. It is important to examine whether LLMs analyzing patient responses from interviews can outperform traditional self-report scales in depression detection.

The study aims to examine the adverse effect of alexithymia on the accuracy of depression detection using AI and the self-report scale HADS-D. The research objectives are (1) to determine whether higher levels of alexithymia are associated with increased severity of depressive symptoms and (2) to assess how alexithymia moderates the accuracy of both the self-report scale and AI-based depression detection models compared to clinical evaluations, which are typically viewed as the gold standard when assessing depression. The findings are intended to inform the effectiveness of AI-based detection methods, and to explore how patient characteristics, like alexithymia, affect the performance of AI systems in identifying mental health symptoms. Findings indicate that alexithymia is associated with greater depression severity. LLMs trained on clinical interview transcripts detect clinician-rated depression more accurately than the self-report scale for people with alexithymia, and several models also outperform the self-report scale across the full sample. These results suggest AI-based tools improve depression detection in people who struggle to describe their emotions. Further research into other mental health disorders is warranted.

## Methods

### Design

This study utilized LLMs to analyze clinical text data for detecting depression. The text data were collected from clinical interviews conducted with depressive patients and control participants. To examine the adverse effect of alexithymia on detection, we compared the accuracies of the LLMs with a self-report scale in detecting depression among subgroups of alexithymia. We involved 299 participants in the analysis.

### Participants

This cross-sectional study was part of an ongoing digital phenotyping research aiming to characterize major depressive disorder (MDD) among Chinese Cantonese speaking adults aged 18–65. It employed a case-control design^16^. The majority cases with lifetime MDD were recruited from outpatient clinics in a local university-affiliated hospital and the diagnosis was made by the attending psychiatrist. About 22% of the MDD subjects were recruited from the community and their diagnosis was confirmed by the Structured Clinical Interview for DSM-5—Clinician Version (SCID-5-CV) administered by a trained medical researcher^17^. The controls were recruited from both the community and sleep centers who were free from psychiatric disorder based on the SCID-5 interview^17^. Exclusion criteria included voice, speech, and language problems, history of psychiatric disorders other than MDDs, and incompetence in giving written informed consent. Data collection was conducted during 2020 and 2023. Ethical approval was obtained from the Joint Chinese University of Hong Kong-New Territories East Cluster Clinical Research Ethics Committee (Ref No: 2020.492). Informed written consent was obtained from all subjects.

### Clinical measurements

#### Hamilton Depression Rating Scale (HAMD)

The structured interview guide for the HAMD was adopted^18^. The interviews were conducted by a psychiatrist with research background (JC). Each interview took around 15–30 min. The 17-item HAMD consisted of 14 questions which were probed sequentially from H1 to H14 to assess depressive symptoms (while questions H15–H17 were rated based on observation during the interview). In the current study, the current depression status was classified as no depression (scored 0–7) and current depression (≥8)^18^; the Cronbach’s alpha reliability is 0.89.

#### Hospital Anxiety and Depression Scale-Depression subscale (HADS-D)

Hospital Anxiety and Depression Scale-Depression subscale (HADS-D)^19^: HADS-D is a 7-item self-report subscale for assessing depressive symptoms in clinical and community populations. Items are scored based on a 4-point scale (range from 0 to 3). In the current study, the depression status was classified as no depression (scored 0–7) and depression (≥8). The Cronbach’s alpha reliability for the current sample was 0.84.

#### Toronto Alexithymia Scale (TAS-20)

The Toronto Alexithymia Scale (TAS-20) is a 20-item self-report questionnaire with three dimensions, namely, difficulty identifying feelings, difficulty describing feelings, and externally oriented thinking (EOT)^20^. Items are scored based on a 5-point scale from strongly disagree (1) to strongly agree (5). The international cut-off values are as follows: 20–50 for without alexithymia, 51–60 as possible alexithymia, and 61–100 as with alexithymia^21,22^. In the current study, the Cronbach’s alpha reliability was 0.90.

#### Large language models (LLMs) training

LLMs were utilized to detect the presence of depression for binary classification^15^. We employed eight LLMs, including Albert Chinese^23^, BERT Chinese^24^, BERT Multilingual^25^, DistilBERT Multilingual^25^, Electra Chinese^24^, Mental BERT Chinese^26^, Roberta Chinese^24^, and XLNet Chinese^27^. The input for each LLM was a sequence of tokens from the Cantonese text transcripts of the recordings of structured clinical interviews using HAMD with 299 participants. We split the texts into manageable chunks^28^. Each chunk contained 384 tokens and an overlap of 128 tokens from the previous chunk. This approach ensured continuity throughout the text while adhering to the maximum token length that these LLMs can process. The prediction for each chunk was aggregated to determine the final predictive label. The probabilities of the predictions were calculated by averaging the probabilities of all chunks associated with the voted label. For instance, if the voted label is “depression,” the probabilities from all chunks predicted as “depression” were used to calculate the accuracy of the prediction. We implemented a 5-fold stratified cross-validation approach to ensure balanced label distribution for robust validation. For example, in each fold, the dataset was split into training and validation sets with an 80:20 ratio. The text data (*n* = 299) was divided into 5 subsets of data (each *n* = 59–60). For training, four subsets were used in one-fold, while the remaining one subset was used for validation. This process was repeated across the folds, resulting in different combinations of the training and validation datasets. To ensure balanced representation, the subsets were stratified based on the labels of depression (i.e., with and without depression), aiming to have an equal number of cases and controls in each fold. Ultimately, each LLM predicted the depressive label for each of the 299 participants.

The tokenizer of each LLM was employed to tokenize the text data, which was then used for training and evaluation. Sparse Categorical Cross entropy served as the loss function, while accuracy was used as the performance metric. Model training was performed over 30 epochs with a batch size of 8. After training, the model’s performance was evaluated on the validation set. This methodology ensured comprehensive evaluation of the model’s generalization and accuracy in detecting depression. In order to guarantee replicability of the results, a random seed was set for the LLMs. Python 3.7 and Jupyter Notebook were utilized for implementing the LLMs.

### Statistical analysis

The analysis has two parts. Part 1 involves generalized linear models - logistic regression (GLMs) to examine the association between self-report and clinical ratings of depression, including the moderation effect of alexithymia. GLM crude model 1 examines the association between alexithymia groups and depression (based on the HAMD-17 score; as the dependent variable, DV). In addition, to ensure that the token lengths of the text data for LLMs were consistent among the three alexithymia subgroups, we performed a GLM to examine the differences in the average number of tokens among these subgroups, controlling for the depressive label (i.e., with depression or not), sex, and age. GLM adjusted model 1 investigates the main effects of alexithymia groups and self-report ratings (HADS-D), as well as the moderation effects of alexithymia groups x HADS-D, on the depression (DV). In contrast, GLM adjusted models 2–9 focused on the probabilities of LLMs detection of depression instead of HADS-D in its investigation. The statistical analysis is performed using R 4.3.2 with a 95% confidence level and *p* < 0.05. Furthermore, Part 2 focuses on using self-report ratings (HADS-D) and LLMs detection to detect depression using Receiver Operating Characteristic - Area Under the Curve (ROC-AUC) analysis. The AUCs of HADS-D and LLMs detection in detecting depression were measured separately. We assessed these AUCs within each alexithymia group to determine group differences. The ROC-AUC analysis was conducted using the “pROC” package of R, with a 95% confidence level and *p* < 0.05. Furthermore, sensitivity analyses were performed specifically in MDD cases.

## Results

### Characteristics of the participants

Table 1 lists the number of participants in each subgroup. We included 299 participants (i.e., 105 controls and 194 MDD subjects, mean age = 53.33 ± 11.29, female *n* = 171). Based on the HAMD-17 score, 186 (62.2%) participants were without and 113 (37.8%) were with depression. Among the 194 MDD cases, 107 were of unremitted status (i.e., HAMD-17 score ≥8) and 87 were remitted. While based on the HADS-D score, 171 (57.2%) were without and 90 (30.1%) with depression (total valid *n* = 261, missing *n* = 38, 12.7%). Pearson’s correlation analysis indicated a moderate positive correlation between the HAMD-17 and HADS-D scores (*n* = 261, *r* = 0.56, *p* < 0.001). Furthermore, there were 108 (36.1%) of them without, 56 (18.7%) with possible, and 50 (16.7%) with alexithymia (total valid *n* = 214, missing *n* = 85).

**Table 1:** Number of participants with and without depression and alexithymia (*n* = 299)

	Alex	HAMD-No Depression	HAMD-Depression	HADS-D-No Depression	HADS-D-Depression
No Alex	108 (36.12)	91 (48.92)	17 (15.04)	96 (56.14)	12 (13.33)
Possible Alex	56 (18.73)	33 (17.74)	23 (20.35)	31 (18.13)	25 (27.78)
Alex	50 (16.72)	15 (8.06)	35 (30.97)	11 (6.43)	39 (43.33)
Alex Missing	85 (28.43)	47 (25.27)	38 (33.63)	33 (19.3)	14 (15.56)
Total	299 (100)	186 (100)	113 (100)	171 (100)	90 (100)

*Alex* Alexithymia, *HAMD* Hamilton Depression Rating Scale, *HADS-D* Hospital Anxiety and Depression Scale-Depression Subscale.

### Associations of LLMs and HADS-D with clinician-rated depression

Table 2 lists the AUC values for assessing the performance of the HADS-D self-report scale (0.79) and the eight LLMs (0.83–0.89) in detecting depression among all the participants. Table 3 presents the results of the subgroup analysis for the HADS-D and LLMs among three alexithymia groups. The AUCs for the HADS-D were 0.35–0.83: 0.78 in those without alexithymia, 0.83 in those with possible alexithymia, and 0.35 in those with alexithymia. There was no significant difference between those without alexithymia and those with possible alexithymia. The lowest accuracy in detecting depression using the HADS-D was observed among individuals with alexithymia (i.e., AUC = 0.35) compared with those without alexithymia (*p* <0.001). In addition, the GLM results indicated no significant differences in token length of the text data among the subgroups (all ps > 0.05), with means and standard deviations as follows: 306.35 ± 119.17 for those without alexithymia, 364.51 ± 102.61 for those with possible alexithymia, and 386.33 ± 103.60 for those with alexithymia.

**Table 2:** AUC values of HAMD vs. HADSD, and HAMD vs. LLMs

Item	AUC	95% CI	*p*	*p* for HADS-D comparison
**All participants**				
HADS-D	0.788	0.732–0.844	<0.001***	NA
Albert Chinese	0.847	0.800–0.893	<0.001***	0.101
BERT Chinese	0.829	0.781–0.876	<0.001***	0.317
BERT Multilingual	0.869	0.828–0.910	<0.001***	0.013*
DistilBERT Multilingual	0.839	0.794–0.884	<0.001***	0.076
Electra Chinese	0.881	0.841–0.921	<0.001***	0.001**
Mental BERT Chinese	0.869	0.827–0.911	<0.001***	0.008**
Roberta Chinese	0.886	0.847–0.925	<0.001***	0.001**
XLNet Chinese	0.842	0.797–0.887	<0.001***	0.134
**MDD cases only**				
HADS-D	0.705	0.622–0.787	<0.001***	NA
Albert Chinese	0.802	0.738–0.867	<0.001***	0.056
BERT Chinese	0.757	0.687–0.827	<0.001***	0.539
BERT Multilingual	0.819	0.757–0.881	<0.001***	0.026*
DistilBERT Multilingual	0.798	0.735–0.862	<0.001***	0.053
Electra Chinese	0.849	0.794–0.903	<0.001***	0.001**
Mental BERT Chinese	0.834	0.776–0.893	<0.001***	0.007**
Roberta Chinese	0.859	0.806–0.912	<0.001***	<0.001***
XLNet Chinese	0.803	0.740–0.865	<0.001***	0.121

*p* for HADS-D comparison: the *p* value of AUC values of HAMD vs. HADS-D compared with HAMD vs. each LLM.

*AUC* Area under the curve, *HAMD* Hamilton Depression Rating Scale, *HADS-D* Hospital Anxiety and Depression Scale-Depression Subscale, *LLM* Large Language Model, *MDD* Major depressive disorder, *p* *p*-value.

*p < 0.05, **p < 0.01, ***p < 0.001.

**Table 3:** AUC values of HAMD vs. HADS-D, and HAMD vs. LLMs among Alexithymia groups

Item	Without Alex	Possible Alex	With Alex	*p* for Without vs. Possible Alex	*p* for Without vs. With Alex
**All participants**	*n* = 108	*n* = 56	*n* = 50	*n* = 164	*n* = 158
	**AUC**	**AUC**	**AUC**	**AUC**	**AUC**
HADS-D	0.780	0.825	0.351	0.581	<0.001***
Albert Chinese	0.769	0.795	0.848	0.769	0.379
BERT Chinese	0.815	0.784	0.791	0.708	0.786
BERT Multilingual	0.840	0.788	0.956	0.482	0.041*
DistilBERT Multilingual	0.841	0.735	0.819	0.168	0.796
Electra Chinese	0.825	0.810	0.869	0.843	0.619
Mental BERT Chinese	0.840	0.793	0.871	0.558	0.691
Roberta Chinese	0.878	0.810	0.928	0.292	0.331
XLNet Chinese	0.810	0.701	0.880	0.210	0.315
**MDD cases only**	*n* = 45	*n* = 34	*n* = 46	*n* = 79	*n* = 91
	**AUC**	**AUC**	**AUC**	**AUC**	**AUC**
HADS-D	0.711	0.825	0.300	0.310	<0.001***
Albert Chinese	0.710	0.796	0.800	0.448	0.425
BERT Chinese	0.712	0.704	0.735	0.947	0.850
BERT Multilingual	0.788	0.693	0.940	0.399	0.064
DistilBERT Multilingual	0.793	0.693	0.764	0.381	0.797
Electra Chinese	0.795	0.796	0.821	0.989	0.822
Mental BERT Chinese	0.846	0.714	0.826	0.233	0.842
Roberta Chinese	0.830	0.804	0.904	0.786	0.337
XLNet Chinese	0.710	0.707	0.839	0.983	0.204

*AUC* Area under the curve, *HAMD* Hamilton Depression Rating Scale, *HADS-D* Hospital Anxiety and Depression Scale-Depression Subscale, *LLM* Large Language Model, *Alex* Alexithymia, *p* *p*-value.

*p < 0.05, ***p < 0.001.

On the other hand, four out of eight LLMs achieved better AUCs than the HADS-D, and all LLMs showed no significant differences in AUCs among the three alexithymia groups. As shown in Table 2, LLMs achieved AUCs of 0.83–0.89 in all participants (*n* = 299), with AUCs of 0.77–0.88, 0.70–0.81, and 0.79–0.96 in the three alexithymia subgroups, respectively. The AUCs of four out of eight LLMs (AUCs = 0.87–0.89) were significantly higher than that of HADS-D (AUC = 0.79), all ps < 0.05. The four LLMs were BERT Multilingual (AUC = 0.87, *p* = 0.013), Electra Chinese (AUC = 0.89, *p* = 0.001), Mental BERT Chinese (AUC = 0.87, *p* = 0.008), and Roberta Chinese (AUC = 0.89, *p* = 0.001). In addition, in the MDD case-only sensitivity analysis, the aforementioned four LLMs maintained a higher AUC compared to HADS-D (0.82–0.86 vs. 0.71, all ps < 0.05). Moreover, as shown in Table 3, the AUCs of LLMs between individuals without alexithymia and those with possible alexithymia (0.71–0.85 vs. 0.69–0.80, all ps > 0.05), and between individuals without alexithymia and those with alexithymia (0.71–0.85 vs. 0.74–0.94, all ps > 0.05), did not have any significant differences. This suggests that the LLMs’ performance in detecting depression was consistent across individuals with and without alexithymia. There was a significant difference in the AUCs of HADS-D between individuals without alexithymia and those with alexithymia (0.78 vs. 0.35, *p* < 0.001), indicating that using self-reporting scales to identify depression in individuals with alexithymia may lead to inaccurate results. Nevertheless, there was no significant difference between individuals without alexithymia and those with possible alexithymia (0.78 vs. 0.82, *p* = 0.58). Overall, the results suggested better performance of LLMs than self-report scales.

### Associations between clinician-rated depression and alexithymia

Table 4 reports the result of the GLM crude model 1, examining the associations between alexithymia groups and clinician rated depression (i.e., the HAMD depressive labels). Compared to those without alexithymia, those with possible (*b* = 1.36, 95% CI = 0.61–2.14, *p* < 0.001) and with alexithymia (*b* = 2.54, 95% CI = 1.77–3.38, *p* < 0.001) were associated with a higher likelihood of having depression. The sensitivity analysis conducted specifically on MDD cases yielded similar results. The result provided evidence indicating that higher degrees of alexithymia were associated with a higher chance of getting depression.

**Table 4:** The crude and the adjusted models of GLM—logistic regression association between HAMD and alexithymia, and the moderations of alexithymia x HADS-D on HAMD (*n* = 214)

Crude model 1	*b* (95% CI)	*p*
Possible Alex	1.36 (0.61–2.14)	<0.001***
Alex	2.54 (1.77–3.38)	<0.001***
**Adjusted model 1**	***b (95% CI)***	***p***
Possible Alex	0.13 (−0.89 to 1.09)	0.788
Alex	2.28 (1.26–3.39)	<0.001***
HADS-D scores	0.30 (0.13–0.49)	<0.001***
Possible Alex × HADS-D scores	0.07 (−0.19–0.35)	0.595
Alex × HADS-D scores	−0.37 (−0.62 to −0.14)	0.002**

The numeric moderator (i.e., HADS-D scores) in the models were centered. Reference groups: for HADS-D = without depression; for alexithymia groups = without alexithymia. Controlled for sex and age.

*GLM* generalized linear model, HAMD Hamilton Depression Rating Scale, HADS-D Hospital Anxiety and Depression Scale-Depression Subscale, Alex Alexithymia, *p* *p*-value.

**p < 0.01, ***p < 0.001.

### Moderations of alexithymia, HADS-D, and LLMs

**Fig. 1:**
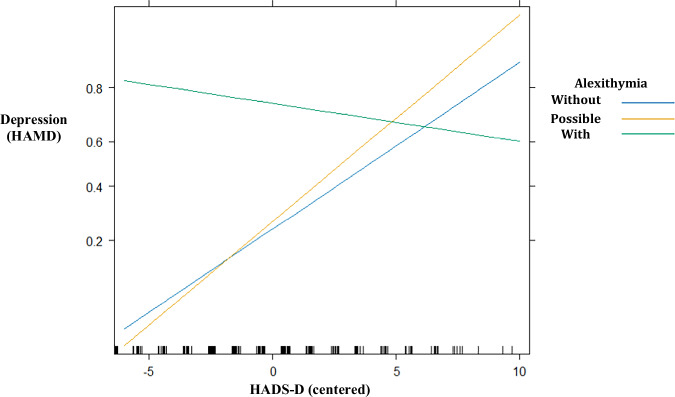
Interaction plot of Alexithymia x HADS-D on depression (HAMD). HAMD Hamilton Depression Rating Scale, HADS-D Hospital Anxiety and Depression Scale-Depression Subscale. The adjusted generalized logistic regression model (*n* = 214) shows a significant negative association between HADS‑D scores and depressive labels (HAMD) in individuals with alexithymia (green line), but not in individuals with possible alexithymia (yellow line). The reference group is individuals without alexithymia (blue line). The model controlled for alexithymia group, HADS‑D scores, sex, and age.

Table 4 also presents the results of the GLM adjusted model 1, examining associations between the HAMD depressive labels as the DV and the main effects of alexithymia groups and the HADS-D self-report depressive scores. Additionally, we explored the moderation effects of alexithymia × HADS-D. There was a positive association found between HADS-D scores and HAMD depressive labels (*b* = 0.30, 95% CI = 0.13–0.49, *p* = 0.001). As in Table 4, Tables 5, 6 present the results of the GLM adjusted models 2–9, examining associations between the HAMD depressive labels as the DV and the main effects of alexithymia groups and the probabilities of AI detection in predicting depressive labels (i.e., with and without depression measured by HAMD) in all LLMs were positively associated with depressive scores (measured by HAMD) (all ps < 0.05). Moreover, in the adjusted model 1 shown in Table 4, the interaction between alexithymia groups and HADS-D scores revealed that compared with individuals without alexithymia, those with alexithymia were less capable of detecting depression (*b* = −0.37, 95% CI = −0.62 to −0.14, *p* = 0.002). In other words, individuals with alexithymia had less capability to accurately rate their depressive symptoms through self-report measures (HADS-D) compared to those without alexithymia. Figure 1 plots this interaction for visualization. Interestingly, this interaction effect was not observed in individuals with possible alexithymia. In contrast, in the adjusted models 2–9 shown in Tables 5, 6, the interaction effect of alexithymia and LLM probabilities of depression was not observed (all ps > 0.05), indicating that LLM detection of depression was not affected by the presence of alexithymia. The sensitivity analysis conducted on MDD cases only yielded similar results. In addition, when conducting the sensitivity analysis using TAS scores instead of alexithymia subgroups, similar results were obtained. These findings highlighted the influence of alexithymia on the accuracy of self-rated depression.

**Table 5:** The adjusted models of GLM—logistic regression association between HAMD and alexithymia, and the moderations of alexithymia x LLM on HAMD (*n* = 214)

	2. Albert Chinese		3. BERT Chinese		4. BERT Multilingual		5. DistilBERT Multilingual	
Adjusted Models 2–9	*b* (95% CI)	*p*	*b* (95% CI)	*p*	*b* (95% CI)	*p*	b (95% CI)	*p*
Possible Alex	1.09 (0.19–1.98)	0.016*	1.23 (0.39–2.08)	0.004**	1.17 (0.24–2.13)	0.014*	1.34 (0.52–2.19)	0.002**
Alex	1.84 (0.81–2.85)	<0.001***	2.01 (1.09–2.95)	<0.001***	1.99 (0.65–3.33)	0.002**	2.05 (1.07–3.06)	<0.001***
LLM probabilities of depression	3.09 (1.41–5.03)	<0.001***	2.34 (1.14–3.63)	<0.001***	3.21 (1.88–4.77)	<0.001***	2.10 (0.93–3.39)	<0.001***
Possible Alex × LLM probabilities of depression	0.20 (−2.35 to 2.79)	0.877	−0.51 (−2.25 to 1.25)	0.567	−1.04 (−3.06 to 0.95)	0.301	−0.85 (−2.57 to 0.84)	0.327
Alex × LLM probabilities of depression	1.17 (−1.59 to 4.16)	0.415	0.28 (−1.64 to 2.27)	0.778	2.67 (−0.01 to 5.96)	0.070	1.36 (−0.66 to 3.52)	0.196

The numeric moderator (i.e., LLM probabilities of depression) in the models were centered. Reference groups: for alexithymia groups = without alexithymia. Controlled for sex and age.

*GLM* generalized linear model, *HAMD* Hamilton Depression Rating Scale, *LLM* Large Language Model, *Alex* Alexithymia, *p* *p*-value.

*p < 0.05, **p < 0.01, ***p < 0.001.

**Table 6:** The adjusted models of GLM—logistic regression association between HAMD and alexithymia, and the moderations of alexithymia x LLMs on HAMD (*n* = 214)

	6. Electra Chinese		7. Mental BERT Chinese		8. Roberta Chinese		9. XLNet Chinese	
Adjusted Models 2–9	*b* (95% CI)	*p*	*b* (95% CI)	*p*	*b* (95% CI)	*p*	*b* (95% CI)	*p*
Possible Alex	1.05 (0.15–1.95)	0.022*	0.98 (0.10–1.85)	0.028*	1.34 (0.48–2.24)	0.003**	1.12 (0.33–1.93)	0.006**
Alex	1.68 (0.69–2.67)	<0.001***	1.78 (0.77–2.80)	<0.001***	1.59 (0.42–2.71)	0.005**	1.85 (0.89–2.80)	<0.001***
LLM probabilities of depression	3.11 (1.76–4.58)	<0.001***	2.38 (1.19–3.64)	<0.001***	2.81 (1.54–4.20)	<0.001***	1.77 (0.59–2.98)	0.003**
Possible Alex × LLM probabilities of depression	−0.15 (−2.14 to 1.89)	0.883	−0.20 (−1.92 to 1.56)	0.819	−0.58 (−2.44 to 1.30)	0.539	−0.93 (−2.57 to 0.73)	0.267
Alex × LLM probabilities of depression	0.12 (−1.95 to 2.28)	0.911	1.19 (−0.80 to 3.37)	0.257	1.66 (−0.59 to 4.20)	0.167	1.22 (−0.69 to 3.27)	0.225

The numeric moderator (i.e., LLM probabilities of depression) in the models were centered. Reference groups: for alexithymia groups = without alexithymia. Controlled for sex and age.

*GLM* generalized linear model, *HAMD* Hamilton Depression Rating Scale, *LLM* Large Language Model, *Alex* Alexithymia, *p* *p*-value.

*p < 0.05, **p < 0.01, ***p < 0.001.

## Discussion

This study compares the performance of a self-report scale with AI-based methods for detecting depression in individuals with alexithymia. The research evaluated the performance of the self-report scale HADS-D in assessing clinician-rated depression, revealing its inaccuracy, as indicated by a low AUC of 0.35. This suggests that alexithymia diminishes the reliability of self-report scales, making them less effective for depression detection compared to individuals without the condition. The available neural evidence supports the current findings, suggesting that individuals with alexithymia tend to exhibit less accurate self-reporting of depressive symptoms. For example, prior neuroimaging studies have indicated that individuals with higher levels of alexithymia tend to have smaller volumes in key brain regions involved in emotion perception and experience, including the left insula, left amygdala, orbital frontal cortex and striatum^29^. Research also indicates that patients with both MDD and higher levels of alexithymia exhibited decreased gray matter volume in the fusiform gyrus, a region responsible for processing faces^30^ and the relevant emotion recognition^31^.

Conversely, the current results indicate that LLMs trained with clinical interview text data exhibit strong performance in identifying clinician-rated depression, particularly for individuals with alexithymia. While linguistic features are crucial in the AI-driven detection of emotions and mental health symptoms^8,9^, we demonstrated that LLMs achieved satisfactory AUCs across the three groups with and without alexithymia. Moreover, these findings suggest that AI models enhance assessment accuracy in individuals with alexithymia compared to traditional self-report methods. In particular, our results showed that there was a positive association between alexithymia and depression severity, which aligns with the previous evidence^6^. It is speculated that alexithymia may pose a significant challenge in detecting depression. Consequently, applying AI techniques to analyze patient text data for mental disorder detection is expected to become a significant focus in digital and precision medicine^32^.

Clinician evaluations are important because they begin with patient self-reports but are significantly improved by the clinician’s expertise and evidence-based practice. Clinicians may employ clinical acumen and sometimes assist with well validated assessment instruments to interpret patient responses, using follow-up questions to refine their understanding, resulting in more nuanced and accurate evaluations. The interactive nature of clinical interviews allows clinicians to explore patients’ emotions and symptoms more thoroughly, addressing biases that may arise from self-reporting. This makes clinician assessments particularly valuable, often positioning clinical interviews as the gold standard for assessing depression. However, the long waiting times and often limited availability and accessibility of trained clinicians and medical assessments, including psychiatric interviews^33^, highlight the need to prioritize the development of automated mental health assessments. The current findings indicate that AI models can enhance the accuracy of guided-AI assessments, such as interactive AI chatbots and AI-assisted information extraction tools in mental health. LLMs have been employed for various mental health tasks, such as targeted information extraction, human-computer interaction, content generation (also known as generative AI), and logical reasoning that simulates human judgment^34,35^. These models have the potential to enhance psychiatric assessments by improving performance and reducing reliance solely on self-report scales for depression screening. Developing advanced and accurate algorithms to detect depression is emerging research^10,36^. Implementing AI-powered systems, either in automated or semi-automated forms, to gather data for both clinician and AI assessments could be a promising path for advancement.

The study examines how alexithymia affects the detection of depression, with potential implications for detecting other mental health disorders. Deficits in processing emotions, such as alexithymia, can impact the recognition and expression of emotions^4,6^, suggesting similar effects may extend to disorders like anxiety and post-traumatic stress disorder. Further investigation is needed to understand how emotional deficits affect AI detection across various mental health disorders. Factors such as personality traits, cultural background, education level, socioeconomic status, and communication skills can influence symptom reporting in both positive and negative ways. Understanding these factors can enhance our understanding of how AI applications can effectively detect mental health conditions^17^. Individuals with alexithymia may face challenges in accessing emotional support from others^37^. Further research can explore how AI technologies, such as chatbots, can offer interactive assessments for individuals with alexithymia, considering their emotional processing limitations.

Our study has several limitations that future research should address. First, we used the TAS, a self-report scale, to assess alexithymia. Employing a more rigorous assessment method, such as the Toronto Structured Interview for Alexithymia^8^, could provide a more thorough evaluation of alexithymia. Second, comparing the HAMD and HADS-D poses certain challenges, especially in groups with alexithymia. The HAMD includes somatic symptoms like sleep, appetite, and fatigue, which might be less influenced by alexithymia, whereas the HADS-D primarily addresses thoughts and feelings. Self-report scales rely on pre-designed items, which might restrict their ability to accurately capture symptoms. While it is worth noting that self-report scales consist of pre-designed items, which may limit their ability to capture symptoms comprehensively. Despite this limitation, the present comparison between the groups with and without alexithymia remains valid. We used the same set of scales, i.e., HAMD and HADS-D, across the three groups of Alexithymia. Thus, the difference between HAMD and HADS-D will not affect the comparison results and conclusion. Future studies could compare AI models with a broader range of self-report scales. Third, there was missing data for the alexithymia scale (*n* = 85, 28.43%), which was excluded from the analysis when evaluating model performance in the subgroup analyses. Finally, the AI models were trained using transcript text from clinical interviews of the HAMD, while the HAMD score was the primary outcome of this study. Beyond internal cross-validation, future research may train AI models with data collected from unstructured clinical interactions, such as medical consultations, and participants’ free speech. This will be followed by an independent clinical evaluation to externally validate the performance of the models.

In conclusion, this study suggests that self-report scales are less effective at detecting depression in individuals with alexithymia, while AI models can accurately identify depression in these cases by studying clinical interview data. By incorporating a broad spectrum of patient characteristics, researchers can deepen their understanding of mental health detection using AI models. This comprehensive approach allows for an evaluation of both the positive and negative effects of patient characteristics on AI performance, ultimately improving the generalizability and accuracy of AI-based detection of mental disorders. The findings highlight the crucial role that individual patient characteristics play in affecting the accuracy of depression detection. This has significant implications for enhancing the effectiveness of AI-based mental health detection by considering the unique attributes of each patient, paving the way for future advancements in personalized medicine.

## Supplementary information

Description of Additional Supplementary files

Supplementary Data 1

## Data Availability

Confidential information from participants cannot be shared. The source data for Fig. 1 is in Supplementary Data 1.
